# Risk factors for postoperative febrile urinary tract infection in patients with urolithiasis: a meta-analysis

**DOI:** 10.3389/fsurg.2026.1772261

**Published:** 2026-03-02

**Authors:** Zimei Mo, Puzhao Liang, Yongtong Ruan

**Affiliations:** Yangjiang Hospital of Traditional Chinese Medicine, Affiliated with Guangzhou University of Chinese Medicine, Yangjiang, China

**Keywords:** febrile urinary tract infection (FUTI), meta-analysis, postoperative, risk factors, urinary calculi surgery

## Abstract

**Objective:**

To identify risk factors for febrile urinary tract infection (FUTI) following surgical intervention for urinary stones.

**Methods:**

We systematically searched seven databases (from CNKI to EMBASE) from inception to May 2025 for cohort and case-control studies investigating risk factors for FUTI. Two investigators independently screened studies, extracted data, and assessed quality (Newcastle-Ottawa Scale). Adjusted odds ratio (OR) with 95% confidence interval (CI) were used as effect measures. Meta-analysis was performed using Stata 12.0.

**Results:**

16 studies (*n* = 5,366) revealed FUTI incidence of 17% (95%*CI*:12.6–21.3%). Ten significant risk factors were identified: (1) General factors: Stone size (*OR* = 1.29, 95%*CI*:1.09–1.52) and operative duration (*OR* = 1.05, 95%*CI*:1.01–1.10). (2) Comorbidity: Diabetes (*OR* = 2.18, 95%*CI*:1.65–2.87), Renal insufficiency (*OR* = 3.19, 95%*CI*:2.16–4.70). (3) Preoperative: preoperative hydronephrosis (*OR* = 2.33, 95%*CI*: 1.14–4.76), elevated preoperative procalcitonin (*OR* = 1.08, 95%*CI*: 1.03–1.13), preoperative pyuria (*OR* = 4.05, 95%*CI*:1.88–8.74), preoperative bacteriuria (*OR* = 2.45, 95%*CI*: 2.07–2.90), perinephric fat stranding (*OR* = 5.09, 95% *CI*:1.71–15.14), and tissue margin sign (*OR* = 2.84, 95%*CI*:1.91–4.23).

**Conclusion:**

Diabetes mellitus, renal insufficiency, preoperative hydronephrosis, elevated procalcitonin, preoperative pyuria, preoperative bacteriuria, perinephric fat stranding, tissue rim sign, operative duration, and stone size are potential independent predictors of FUTI after urinary stone surgery. These findings enable targeted prevention strategies for high-risk urolithiasis patients.

## 1 Introduction

Urolithiasis, a common urological disorder with a global prevalence of approximately 10% (1), can lead to a series of clinical manifestations, including renal colic, hematuria, and urinary tract infections (UTIs). Currently, minimally invasive procedures are the primary treatment approach. With advancements in technology, the efficacy of minimally invasive surgery for urolithiasis has been widely recognized, demonstrating fewer complications and high stone clearance rates. However, as its application increases, the incidence of postoperative complications has also risen. Common complications include fever, hematuria, and infection, while more severe but rare complications encompass obstruction and urosepsis. Recent data have shown that postoperative UTI is influenced by multiple peri-operative factors, including operative time, stone burden and ureterorenoscope type (2). FUTI is one of the most frequent postoperative complications of urolithiasis. FUTI was defined as fever (>38 °C) accompanied by pyuria within one week postoperatively, after excluding other infectious etiologies. It may lead to severe conditions such as sepsis, septic shock, and even life-threatening outcomes (3, 4). Identifying risk factors is crucial for preventing infectious complications and mitigating associated risks. Although several studies have sought to identify risk factors for postoperative infectious complications in patients with urolithiasis, the evidence remains inconsistent (5–7). For instance, Cagdas Sene et al. (8) identified hydronephrosis, a history of UTIs, and elevated urinary white blood cell counts as significant independent predictors of FUTI, whereas Shigeki Koterazawa et al. (9) reported female sex, diabetes mellitus, and prolonged operative time as risk factors. Due to the variability and specificity among studies, systematic evidence regarding FUTI risk factors remains insufficient. This study employs a systematic review and meta-analysis to comprehensively evaluate the major factors influencing the occurrence of FUTI following minimally invasive surgery for urolithiasis. The findings aim to provide clinicians with valuable insights for risk stratification, personalized perioperative management, and targeted interventions to reduce the incidence of UTIs and improve patient outcomes.

## 2 Materials and methods

This meta-analysis was conducted in accordance with the Preferred Reporting Items for Systematic Reviews and Meta-Analyses (PRISMA) guidelines (10). The study was registered on the PROSPERO platform (Registration No: CRD420251060966).

### 2.1 Literature inclusion and exclusion criteria

FUTI was defined as fever (>38 °C) accompanied by pyuria within one week postoperatively, after excluding other infectious etiologies. the one-week postoperative period was selected to enhance the causal linkage between the infection and the surgical procedure itself. This interval corresponds to the phase of maximal local tissue trauma and transient immunosuppression, during which microorganisms potentially introduced during surgery are most likely to manifest clinically. Furthermore, we clarify that this timeframe is intended to specifically capture infections related to the index operation (e.g., from irrigation, mucosal injury, or manipulation of colonized urine), while minimizing the inclusion of later-onset events that are more likely associated with indwelling stent biofilm or community-acquired infections after discharge.

Inclusion criteria:

(1) Study designs limited to cohort studies or case-control studies;
(2) Study population clearly defined as patients subjected to surgical treatment for urinary tract calculi;
(3) Participants aged ≥18 years, with no gender restrictions;
(4) Studies must explicitly report risk factors associated with FUTI following urinary tract calculi surgery.

Exclusion criteria:

(1) Studies involving animal experiments, conference abstracts, case reports, or review articles;
(2) Duplicate publications or studies with a NOS score ≤4;
(3) Studies failing to clearly specify participant sources, screening procedures, or inclusion/exclusion criteria;
(4) Studies with missing or incomplete key raw data;
(5) Studies not published in Chinese or English.

### 2.2 Literature search strategy

We conducted a systematic search of seven databases—CNKI, VIP, WanFang Data, Cochrane Library, PubMed, EMBASE, and CBM—for studies examining risk factors for FUTI after urolithiasis surgery. The search timeframe spanned from the inception of each database to May 7, 2025. Both Medical Subject Headings (MeSH) and free-text terms were employed. The Chinese search terms comprised: “postoperative urolithiasis”, “post-ureteroscopic lithotripsy”, “post-percutaneous nephrolithotomy”, “risk factors” “risk factor analysis”, “febrile urinary tract infection”, and “urinary tract infection-associated fever”. The English search terms included: “Urolithiasis Surgery,” “Endoscopic Stone Surgery,” “Urinary Tract Infections,” “Infections, Urinary Tract,” “Infection, Urinary Tract,” “Tract Infections, Urinary,” “Tract Infection, Urinary,” “Urinary Tract Infection,” “Febrile,” “Risk Factors,” “Risk factor,” and “Factor, Risk.” Filters were applied for English-language publications, human studies, and adults (aged 18 years or older). The detailed search strategy for PubMed is presented in Table 1. The complete search strategy is available in Supplementary Material 1. Additionally, manual searches of the reference lists of included studies were performed to identify additional eligible literature.

**Table 1:** Pubmed search strategy.

No.	Search results
#1	([“Urinary Tract Infections”(MeSH Terms)] OR [Urinary tract infection(MeSH Terms)] OR [Infections, Urinary Tract(MeSH Terms)] OR [Infection, Urinary Tract(MeSH Terms)] OR [Tract Infections, Urinary(MeSH Terms)] OR [Tract Infection, Urinary(MeSH Terms)] OR [Urinary Tract Infections(Title/Abstract)] OR [Urinary Tract Infection(Title/Abstract)] OR [Infections, Urinary Tract(Title/Abstract)] OR [Infection, Urinary Tract(Title/Abstract)] OR [Tract Infections, Urinary(Title/Abstract)] OR [Tract Infection, Urinary(Title/Abstract)])
#2	[Febrile(Title/Abstract)]
#3	([Urolithiasis Surgery(Title/Abstract)] OR [Endoscopic Stone Surgery(Title/Abstract)])
#4	([“Risk Factors”(MeSH Terms)] OR [Risk factor(MeSH Terms)] OR [Factor, Risk(MeSH Terms)] OR [Risk Factors(Title/Abstract)] OR [Risk factor(Title/Abstract)] OR [Factor, Risk(Title/Abstract)])
#5	#1 AND #2 AND #3 AND #4

### 2.3 Literature screening and data extraction

The initially retrieved literature citations were imported into EndNote software for automatic removal of duplicate records. The remaining articles were independently reviewed in full text by two researchers, with screening strictly based on predefined inclusion and exclusion criteria. Any discrepancies were resolved through adjudication by a third investigator. The final screening results underwent cross-verification by both researchers. Inter-rater agreement was assessed using the Kappa statistic. The extracted data items included: first author's name, year of publication, country of study, study design type (cohort/case-control), total sample size, incidence of postoperative FUTI, and analyzed risk factors. To enable a comprehensive assessment of study context and potential effect modifiers, the following supplementary data were also extracted from each publication: (1) perioperative antibiotic management (routine urine culture, management of positive cultures, type/timing/duration of prophylactic antibiotics); (2) surgical characteristics (procedure type, stone location, technical parameters); and (3) the verbatim definition of FUTI as reported in the source.

### 2.4 Quality assessment of included studies

The methodological quality of the included studies was evaluated using the Newcastle-Ottawa Scale (NOS). This scale consists of eight items across three domains, with a maximum score of 9 points. Based on the scoring criteria, studies were classified as follows: ≤4 points indicated low quality, 5–6 points indicated moderate quality, and ≥7 points indicated high quality. Only studies rated as moderate or high quality were included in this meta-analysis.

### 2.5 Statistical analysis

Statistical analysis was performed using Stata 12.0 software. The adjusted odds ratio (OR) and its 95% confidence interval (CI) were selected as the effect measures. Heterogeneity among studies was assessed using the Q-test combined with the *I^2^* statistic: if *P* ≥ 0.10 and *I^2^* ≤ 50%, it indicated no significant heterogeneity, and a fixed-effects model was applied for synthesis; if *P* < 0.10 or *I^2^* > 50%, significant heterogeneity was assumed, and a random-effects model was employed for pooled analysis. Sensitivity analysis was performed using the leave-one-out method or by comparing the differences between pooled results obtained from fixed-effects and random-effects models to evaluate the robustness of the findings. Subgroup analysis was conducted to explore potential sources of heterogeneity. For risk factors with ≥10 included studies, potential publication bias was examined using funnel plots combined with Egger's regression test. The statistical significance level was set at *P* < 0.05.

## 3 Results

### 3.1 Literature screening process and results

**Figure 1:**
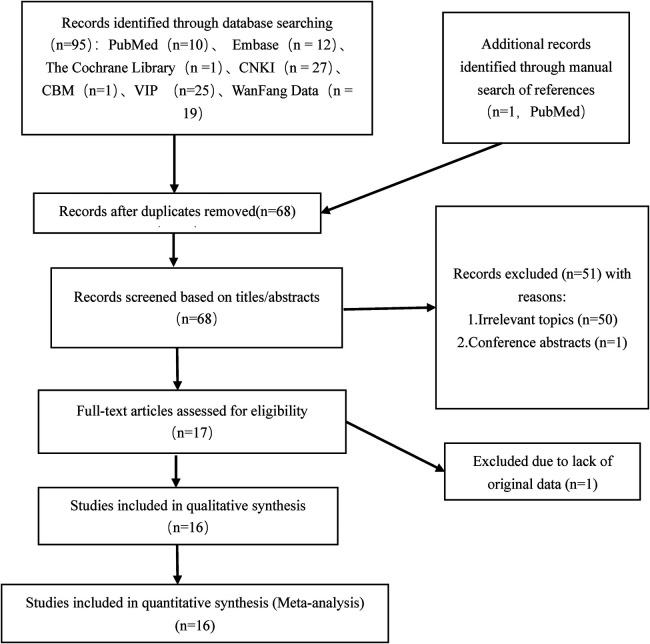
Literature screening process.

A total of 95 articles were identified through database searches. One additional study meeting the inclusion criteria was identified by manually screening the reference lists of included studies. After removing 28 duplicate records, the titles and abstracts of the remaining 68 articles were screened. Among these, 51 were excluded due to irrelevance to the research topic or being conference reports only. The full texts of the remaining 17 articles were assessed for eligibility, and one was further excluded due to missing key raw data. Ultimately, 16 articles met the inclusion criteria and were included in this meta-analysis. The detailed literature screening process is illustrated in Figure 1. Inter-rater agreement was assessed using the Kappa statistic. The results demonstrated almost perfect agreement at both stages (title/abstract screening Kappa = 0.92; full-text screening Kappa = 1.00). Detailed results are presented in Supplementary Tables 1, 2.

### 3.2 Basic characteristics and methodological quality of included studies

Among the 16 studies ultimately included, 8 were Chinese-language publications and 8 were English-language publications. In terms of study design, 12 were case-control studies, while the remaining 4 were cohort studies. A total of 5,366 patients underwent urinary tract stone surgery were included, with postoperative FUTI incidence rates ranging from 0.9% to 65.4%. Sixteen potential risk factors associated with postoperative FUTI were preliminarily identified. Quality assessment using the NOS (Newcastle-Ottawa Scale) demonstrated that all 16 studies scored ≥5 points (range: 5–8), indicating an overall good methodological quality of the included research. Detailed scoring information is provided in Supplementary Table 3. The basic characteristics of each study (e.g., country, study design, sample size, infection rate, analyzed risk factors) along with their respective NOS scores are summarized in Table 2.

**Table 2:** Basic features and literature quality scores of the involved literature.

First author, year	Country	Study design	Sample size (*n*)	Infection rate (%)	Risk factors	NOS score
Mitsuzuka (10) 2016	Japan	Cohort study	153	18.3	Stone size, preoperative ureteral stent placement, gender	7
Itami (11) 2021	Japan	Case-control study	239	13.4	Stone location, maximum Hounsfield unit value, operative duration	7
Lin (12) 2021	China	Case-control study	232	0.9	Preoperative pyuria	8
Koterazawa (8) 2024	Japan	Cohort study	1,626	4.2	Diabetes, stone size, operative duration, gender	7
Kim (13) 2020	South Korea	Case-control study	182	14.3	Stone size, perinephric fat stranding, tissue rim sign	8
Kim (14) 2018	South Korea	Cohort study	304	14.1	Diabetes, renal insufficiency, stone size, preoperative ureteral stent placement, stone location, preoperative hydronephrosis, operative duration, gender, body mass index	7
Kim (15) 2021	South Korea	Cohort study	150	11.3	Stone size, preoperative pyuria, operative duration, preoperative bacteriuria, body mass index	7
Senel (7) 2024	Turkey	Case-control study	511	6.7	Diabetes, preoperative hydronephrosis, urinary white blood cell count	8
Xu (16), 2024	China	Case-control study	253	15.0	Preoperative bacteriuria, gender	8
Ma (17), 2023	China	Case-control study	195	12.3	Diabetes, renal insufficiency, stone size, maximum Hounsfield unit value, operative duration, perinephric fat stranding, tissue rim sign	7
Zhou (18), 2024	China	Case-control study	122	28.7	Diabetes	8
Qian (19), 2023	China	Case-control study	176	16.5	Diabetes	7
Zhang (20), 2022	China	Case-control study	354	20.1	Operative duration, urinary white blood cell count, perinephric fat stranding, tissue rim sign	7
Lu (21), 2021	China	Case-control study	501	13.8	Diabetes, renal insufficiency, stone size, operative duration, perinephric fat stranding, tissue rim sign	7
Xu (22), 2024	China	Case-control study	130	65.4	Diabetes, stone size, stone location, elevated preoperative procalcitonin, operative duration, preoperative bacteriuria, urinary white blood cell count	6
Chen (23), 2024	China	Case-control study	280	27.9	Diabetes, renal insufficiency, stone size, elevated preoperative procalcitonin, operative duration	7

NOS, Newcastle-Ottawa Scale. Risk Factors: Diabetes; Renal insufficiency (impaired renal function); Stone size (kidney stone diameter); Preoperative ureteral stent placement; Stone location (anatomical position of kidney stone); Maximum Hounsfield unit value (peak CT density measurement of stone); Preoperative hydronephrosis (kidney swelling before surgery); Elevated preoperative (elevated procalcitonin levels before surgery); Preoperative pyuria (presence of white blood cells in urine before surgery); Operative duration; Preoperative bacteriuria (presence of bacteria in urine before surgery); Urinary white blood cell count; Gender (patient sex); Perinephric fat stranding (inflammatory changes in perinephric fat on imaging); Tissue rim sign (CT imaging finding around ureter); Body mass index (weight-to-height ratio).

To elucidate potential sources of heterogeneity and provide a comprehensive context for the pooled estimates, detailed data on perioperative antibiotic management, surgical features, and FUTI definitions were extracted from all included studies (Supplementary Tables 4, 5). Based on the transparency and adherence to guideline recommendations in reporting antibiotic regimens, studies were categorized into two groups: Group A, comprising a single study that explicitly reported a standardized prophylaxis protocol guided by preoperative urine culture results, and Group B, comprising the remaining 15 studies with either unclear, non-standardized, or incompletely reported antibiotic strategies (Supplementary Table 4). A qualitative comparison was performed between the study implementing a explicitly reported, culture-guided antibiotic prophylaxis regimen (Group A: Itami 2021) and those with unclear or non-standardized regimens (Group B: 15 studies). The FUTI incidence in the Group A study was 13%. In contrast, the FUTI incidence among Group B studies exhibited a wide range (0.9%–65.4%), with a median value of approximately 18.5%. While formal statistical subgroup analysis was precluded by the presence of only one study in Group A, this descriptive comparison suggests that standardized perioperative antibiotic management, as exemplified by Itami et al. (12), may be associated with a lower reported incidence of FUTI. This observation underscores the potential importance of adhering to guideline-recommended protocols, though it requires confirmation from future studies with more consistent reporting. Furthermore, reporting of surgical characteristics was inconsistent across studies, with frequent omissions of details regarding operative time definitions, ureteral access sheath use, irrigation strategies, and stenting policies (Supplementary Table 5).

### 3.3 Meta-analysis results

#### 3.3.1 Incidence of febrile urinary tract infection in postoperative patients with urolithiasis

**Figure 2:**
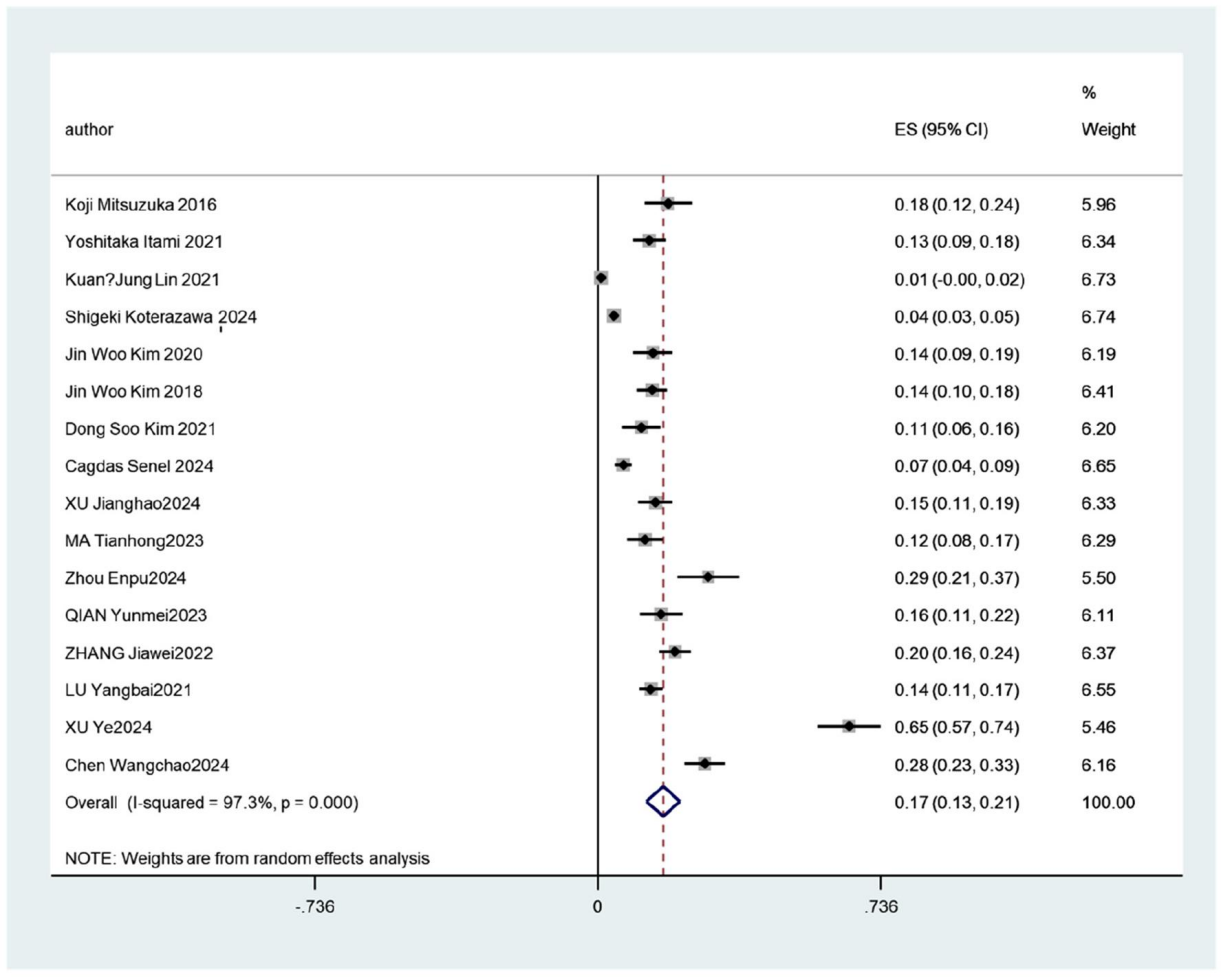
Incidence of febrile urinary tract infection in postoperative patients with urolithiasis. The pooled effect size (incidence rate): 17%, 95% CI (95% confidence interval): 13%–21%.

**Figure 3:**
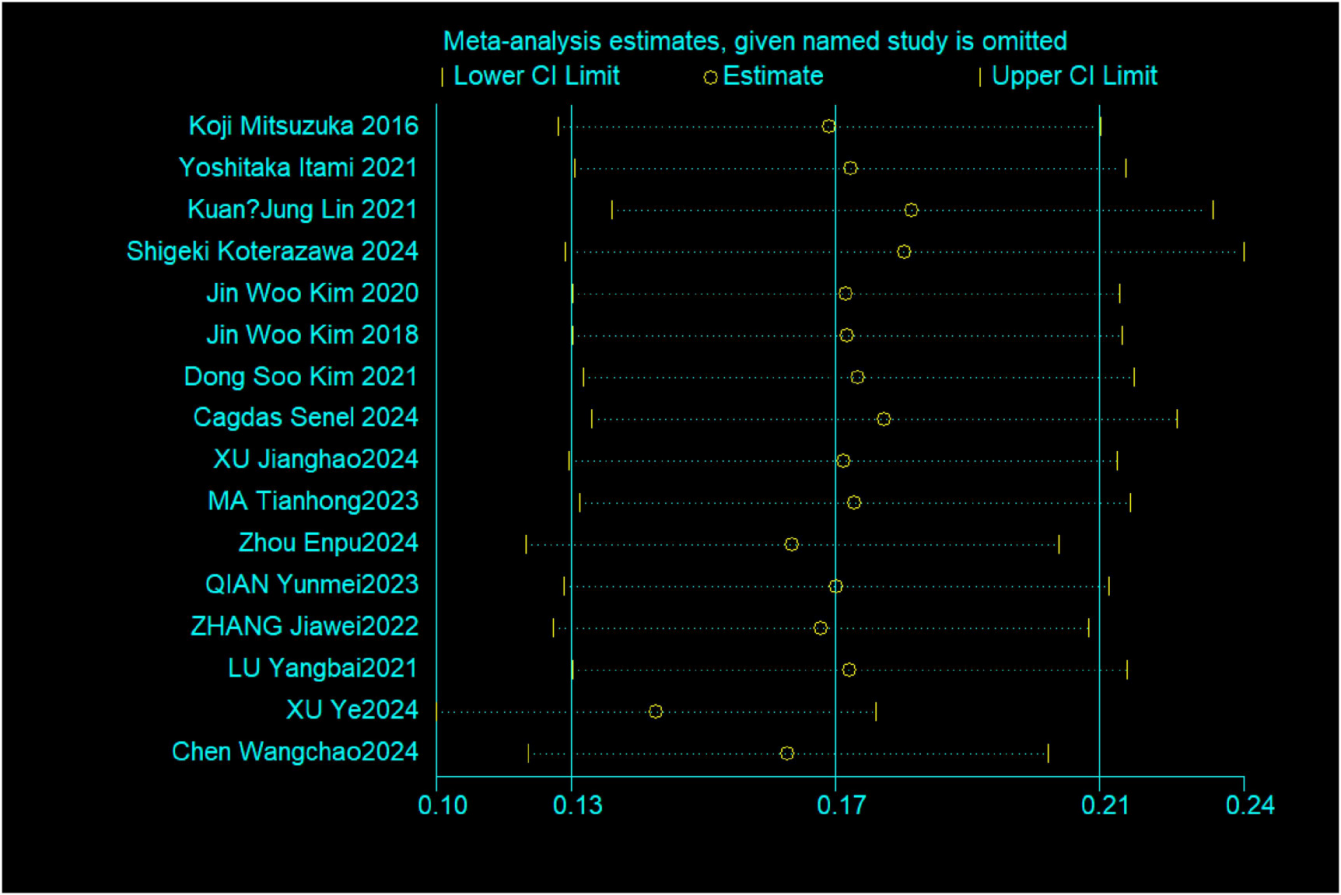
Results of the sensitivity analysis for the incidence of postoperative febrile urinary tract infection in patients with urinary calculi.

**Figure 4:**
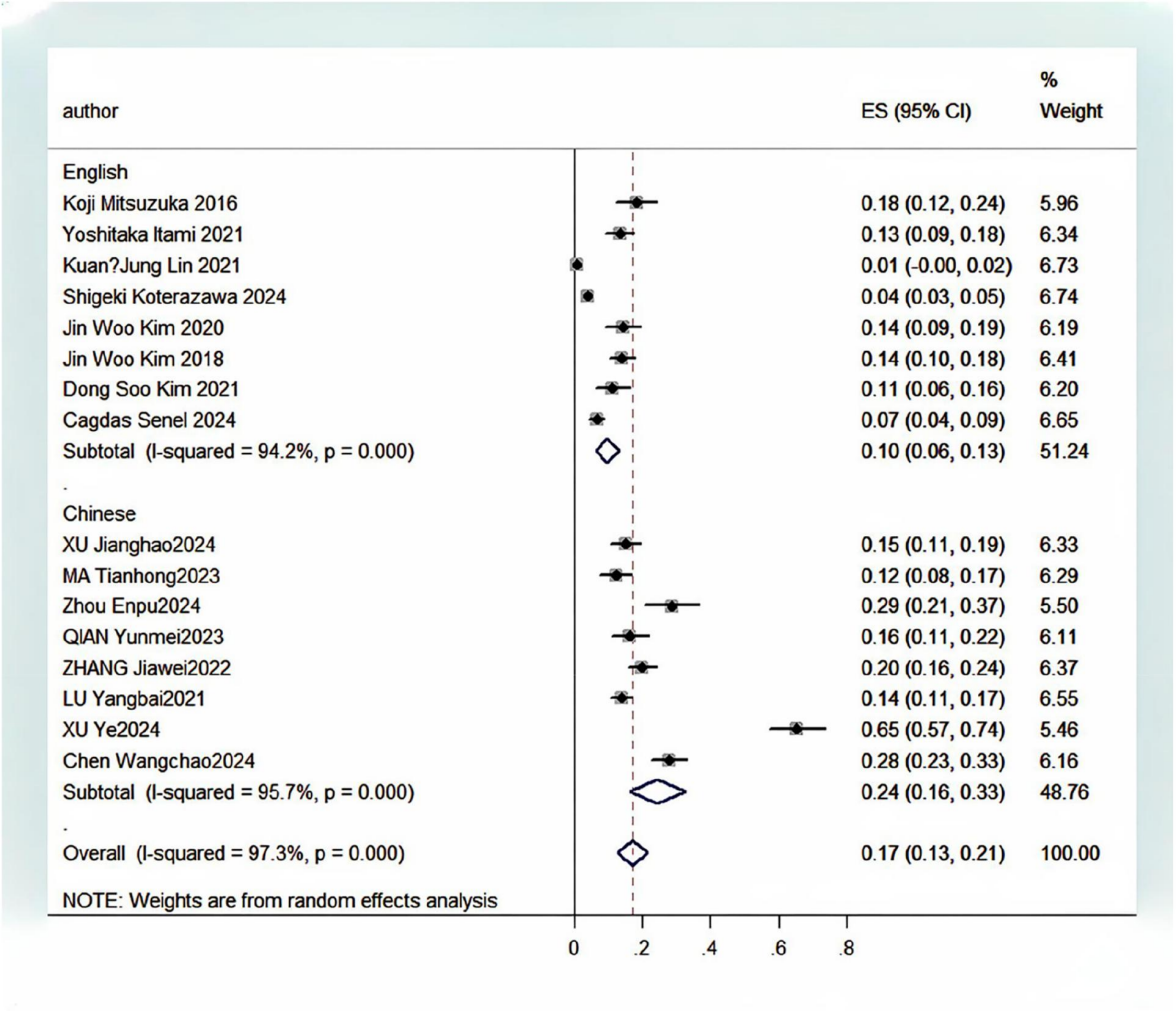
Results of the subgroup analysis for the incidence of postoperative febrile urinary tract infection in patients with urinary calculi.

A significant statistical heterogeneity was observed among the 16 included studies (*I^2^* = 97.3%, *P* < 0.001). A random-effects model was employed for the meta-analysis. The pooled incidence of FUTI after urolithiasis surgery was 17% (95% CI: 12.6%–21.3%), as shown in Figure 2. Given the high heterogeneity in the overall pooled prevalence (*I^2^* = 97.3%, *P* < 0.001), a leave-one-out sensitivity analysis and subgroup analyses were performed to explore potential sources of heterogeneity. After sequentially excluding each study and re-conducting the meta-analysis, the point estimates of the pooled prevalence ranged from 12.6% to 21.3%, and all 95% confidence intervals overlapped with the initial pooled result (17%). This indicates that the overall pooled result is robust and not unduly influenced by any single study, as shown in Figure 3. A subgroup analysis was conducted based on the publication language (Chinese vs. English) of the included studies (Figure 4). The results revealed that the pooled prevalence reported in Chinese-language publications (24%, 95%*CI*: 16%–33%) was significantly higher than that in English-language publications (10%, 95%*CI*: 6%–13%), with a statistically significant between-group difference (*P* < 0.001). However, substantial heterogeneity persisted within each subgroup (Chinese subgroup *I^2^* = 95.7%, English subgroup *I^2^* = 94.2%), indicating the presence of other profound sources of heterogeneity beyond language or regional factors.

#### 3.3.2 General factors

A total of six risk factors were included in the analysis. Among these, stone size (*OR* = 1.29, 95% *CI*: 1.09–1.52, *P* = 0.003) and operative duration (*OR* = 1.05, 95% CI: 1.01–1.10, *P* = 0.031) were significantly associated with postoperative FUTI in patients with urinary tract calculi. In contrast, stone location, maximum Hounsfield unit (HU) value, gender, and body mass index (BMI) showed no statistically significant correlation with postoperative FUTI (Table 3).

**Table 3:** Meta-Analysis results of risk factors for postoperative febrile urinary tract infection in patients with urolithiasis.

Risk factors	Included studies (*n*)	Heterogeneity test		Effect model	OR	95% CI	Z Value	*P* Value
		I^2^ (%)	*P* Value					
Diabetes	9	55.4%	0.022	Random effects model	2.18	1.65–2.87	5.50	0.000
Renal insufficiency	5	0.0%	0.821	Fixed effects model	3.14	2.09–4.71	5.51	0.000
Stone size	6	92.9%	0.000	Random effects model	1.29	1.09–1.52	2.94	0.003
Preoperative ureteral stent placement	2	0.0%	0.509	Fixed effects model	1.57	0.55–4.54	0.84	0.402
Stone location	3	83.7%	0.002	Random effects model	1.59	0.77–3.28	1.26	0.207
Maximum Hounsfield unit value	2	0.0%	0.420	Fixed effects model	1.41	0.70–2.82	0.96	0.336
Preoperative hydronephrosis	2	14.0%	0.281	Fixed effects model	2.33	1.14–4.76	2.30	0.022
Elevated preoperative procalcitonin	2	47.2%	0.169	Fixed effects model	1.08	1.03–1.13	2.96	0.003
Preoperative pyuria	3	0.0%	0.414	Fixed effects model	4.05	1.88–8.74	3.57	0.000
Operative duration	5	91.1%	0.000	Random effects model	1.05	1.01–1.10	2.15	0.031
Preoperative bacteriuria	3	0.0%	0.385	Fixed effects model	2.45	2.07–2.90	10.48	0.000
Urinary white blood cell count	3	98.9%	0.000	Random effects model	0.95	0.37–2.45	0.11	0.913
Gender	4	67.0%	0.028	Random effects model	1.84	0.86–3.94	1.57	0.117
Perinephric fat stranding	4	89.3%	0.000	Random effects model	5.09	1.71–15.14	2.93	0.003
Tissue rim sign	4	36.8%	0.191	Fixed effects model	2.84	1.91–4.23	5.16	0.000
Body mass index	2	0.0%	0.350	Fixed effects model	1.08	0.99–1.18	1.81	0.070

#### 3.3.3 Comorbidity-related factors

Two risk factors were included in the analysis: diabetes mellitus (*OR* = 2.18, 95%*CI*: 1.65–2.87, *P* < 0.010) and renal insufficiency (*OR* = 3.19, 95%*CI*: 2.16–4.70, *P* < 0.010). Both factors demonstrated statistically significant associations with the occurrence of febrile urinary tract infections in patients following urinary stone surgery (Table 3).

#### 3.3.4 Preoperative risk factors

A total of eight risk factors were analyzed, among which six demonstrated statistically significant associations: Preoperative hydronephrosis (*OR* = 2.33, 95%*CI*: 1.14–4.76, *P* = 0.022), Elevated preoperative procalcitonin (*OR* = 1.08, 95%*CI*: 1.03–1.13, *P* = 0.003), Preoperative pyuria (*OR* = 4.05, 95% *CI*: 1.88–8.74, *P* < 0.010), Preoperative bacteriuria (*OR* = 2.45, 95% *CI*: 2.07–2.90, *P* < 0.010), Perinephric fat stranding (*OR* = 5.09, 95%*CI*: 1.71–15.14, *P* = 0.003), Rim sign(*OR* = 2.84, 95%*CI*: 1.91–4.23, *P* < 0.010). In contrast, preoperative ureteral stent placement and urinary white blood cell count showed no statistically significant association with postoperative febrile urinary tract infection in patients with urinary calculi (Table 3).

### 3.4 Sensitivity analysis

A sensitivity analysis was performed by comparing the differences between the pooled results obtained from the fixed-effects model and the random-effects model. The results demonstrated that the pooled effect sizes exhibited significant variability upon switching the effect models for the following factors: stone location, preoperative hydronephrosis, elevated preoperative procalcitonin, operative duration, urinary white blood cell count, and gender, indicating instability in these outcomes. In contrast, no significant differences were observed when the effect models were altered for the following variables: diabetes, renal insufficiency, stone size, preoperative ureteral stent placement, maximum Hounsfield unit value, preoperative pyuria, preoperative bacteriuria, perinephric fat stranding, tissue rim sign, and body mass index, suggesting robust and reliable results (Table 4).

**Table 4:** Sensitivity analysis results of meta-analysis for risk factors of postoperative febrile urinary tract infection in patients with urolithiasis.

Risk factors	Effect model	OR	95% CI	Z value	*P* value
Diabetes	Fixed effects model	1.95	1.69–2.25	9.22	0.000
Renal insufficiency	Random effects model	3.14	2.09–4.71	5.51	0.000
Stone size	Fixed effects model	1.04	1.02–1.06	3.36	0.001
Preoperative ureteral stent placement	Random effects model	1.57	0.55–4.54	0.84	0.402
Stone location	Fixed effects model	1.88	1.59–2.22	7.33	0.000
Maximum hounsfield unit value	Random effects model	1.41	0.70–2.82	0.96	0.336
Preoperative hydronephrosis	Random effects model	2.22	0.93–5.26	1.78	0.075
Elevated preoperative procalcitonin	Random effects model	1.11	0.98–1.25	1.61	0.107
Preoperative pyuria	Random effects model	4.05	1.88–8.74	3.57	0.000
Operative duration	Fixed effects model	1.00	1.00–1.01	1.68	0.093
Preoperative bacteriuria	Random effects model	2.45	2.07–2.90	10.48	0.000
Urinary white blood cell count	Fixed effects model	1.00	1.00–1.01	4.08	0.000
Gender	Fixed effects model	1.89	1.22–2.91	2.87	0.004
Perinephric fat stranding	Fixed effects model	2.43	1.86–3.18	6.50	0.000
Tissue rim sign	Random effects model	2.89	1.70–4.90	3.93	0.000
Body mass index	Random effects model	1.08	0.99–1.18	1.81	0.070

### 3.5 Publication bias

When fewer than 10 studies are included, the ability to effectively detect publication bias is limited, thereby reducing the statistical power of such analyses. Commonly used methods for detecting bias, such as funnel plots or Egger's regression test, may yield false-positive or false-negative results with small sample sizes. Therefore, no further analysis of publication bias was conducted.

## 4 Discussion

Urinary tract calculi are a common condition in urology. Minimally invasive surgery is a safe and effective treatment for urinary tract calculi. Although minimally invasive techniques achieve high stone-free rates with low morbidity, the overall complication rate ranges from 5% to 25%, with infections representing the most frequent complications (25). Postoperative FUTI is one of the most frequently considered significant complications and may even progress to sepsis. This study revealed that the overall prevalence of FUTI following urinary stone surgery was 17%, although this result exhibited substantial heterogeneity (*I^2^* > 97%). Such a degree of heterogeneity is not uncommon in this field and is consistent with previous research contexts (8, 26). The marked heterogeneity primarily stems from considerable variations across clinical centers in terms of patient demographic characteristics, surgical techniques, perioperative management, and most critically, the diagnostic criteria for FUTI—such as the fever threshold (>38 °C vs. >38.5 °C), the requirement for confirmatory urine culture results, and the time window for symptom onset (within 24 h vs. within 1 week postoperatively). The lack of standardization in these diagnostic criteria undoubtedly influences the pooled estimate, representing a key aspect that requires standardization in future research in this field. Sensitivity analysis conducted in this study confirmed the robustness of the pooled results, thereby enhancing the credibility of the main findings.

Sensitivity analysis (Table 4) was crucial for evaluating the reliability of the study findings. The analysis revealed that factors including diabetes, renal insufficiency, preoperative pyuria, preoperative bacteriuria, perinephric fat stranding, tissue rim sign, and stone size exhibited consistent results between the fixed-effect and random-effect models, indicating that these associations were robust and reliable. In contrast, the pooled results for factors such as stone location, preoperative hydronephrosis, preoperative procalcitonin, operative time, urinary white blood cell count, and gender demonstrated significant alterations between different models, suggesting that the reliability of these findings is limited and that caution should be exercised in their clinical interpretation.

The systematically extracted study characteristics (Supplementary Tables 4, 5) provide crucial context for interpreting the considerable heterogeneity observed in this meta-analysis. Notable variability existed in perioperative antibiotic protocols. Only one study (Group A) detailed a guideline-adherent approach involving routine preoperative urine culture with targeted antibiotic therapy. The qualitative comparison suggested a lower FUTI incidence in this study compared to the median incidence among studies with less clearly defined protocols (Group B), highlighting the potentially modifiable role of standardized antibiotic prophylaxis in infection-related outcomes. Although the included studies primarily involved ureteroscopic lithotripsy (URS) or retrograde intrarenal surgery (RIRS) for ureteral or renal stones, meaningful subgroup analysis by surgical technique was precluded due to frequent lack of specific technical parameters. This reporting inconsistency itself constitutes a key finding and underscores that these methodological and clinical variables are likely major contributors to unexplained heterogeneity.

The results of this study indicate that diabetes is a strong predictor of FUTI (*OR* = 2.18). The underlying mechanisms involve impaired immune function due to hyperglycemia—such as suppressed T-cell response and decreased complement activity—microcirculatory disturbances, and an environment conducive to bacterial proliferation. The European Association of Urology (EAU) guidelines also emphasize the importance of enhanced perioperative glycemic control and infection prevention in diabetic patients (27). Renal insufficiency (*OR* = 3.19) represents another robust predictive factor, the pathological mechanism of which may be associated with decreased tubular clearance leading to prolonged retention of calculi and pathogens, thereby facilitating the formation of biofilm-related infections (18). Therefore, preoperative assessment and optimization of renal function are crucial.

Preoperative bacteriuria (*OR* = 2.45) was identified as a key modifiable risk factor. Kutchukian et al. (28) demonstrated that a positive urine culture is an independent correlate of postoperative febrile infections. These findings support the practice of performing preoperative urine cultures and administering targeted antibiotic therapy, which aligns with the EAU guidelines recommending the management of asymptomatic bacteriuria before surgery to reduce postoperative infection risk (27, 29). Similarly, preoperative pyuria (*OR* = 4.05) was also significantly associated with an increased incidence of postoperative FUTI. Although the definition threshold (e.g., ≥5 WBC/HPF vs. ≥10 WBC/HPF) (30) remains controversial across studies (13, 18, 29), its value as a marker of infection and inflammation is well established.

Perirenal space alterations result from fluid release within the bridging septa of perirenal fat, caused by increased lymphatic pressure, inflammation, and edema in the ureteral wall surrounding a renal calculus. The perirenal fat stranding sign is observed in 36%–82% of adult patients with ureteral stones (14). The tissue rim sign arises as a consequence of inflammatory and edematous changes in the ureteral wall, induced by contact with an obstructing ureteral stone; its presence often indicates ureteral wall inflammation and edema (22). According to a study by Jin Woo Kim et al. (14), the tissue rim sign has been reported in 34%–76% of ureteral stone cases and serves as a useful indicator for distinguishing ureteral stones from phleboliths. Therefore, both the perirenal fat stranding and tissue rim signs on non-contrast helical computed tomography reflect inflammatory changes secondary to urinary tract obstruction by calculi. These CT signs provide an objective basis for non-invasive preoperative risk assessment.

Although operative time (*OR* = 1.05) and stone size (*OR* = 1.29) emerged as significant factors in the primary analysis, their high heterogeneity (*I^2^* > 90%) and instability during model transformation suggest that their effects may be moderated by various factors such as surgical technique, stone composition, and irrigation pressure (12, 24). Therefore, they should be regarded as potential risk indicators rather than independent strong determinants for decision-making. Although preoperative procalcitonin (PCT) was statistically significant (*OR* = 1.08, *P* = 0.003), its odds ratio was close to 1.0, indicating a limited absolute effect size and clinical predictive value. Therefore, PCT may be more suitable as a reference indicator in comprehensive assessments rather than serving as an independent decision-making basis.

Based on the robust predictors identified in this study, we propose a targeted perioperative management framework for risk stratification and outcome optimization. First, for patients with diabetes mellitus, strict perioperative blood glucose monitoring and management should be implemented. Urologists should collaborate closely with endocrinologists or primary care physicians to achieve optimal glycemic control (e.g., preoperative HbA1c < 7% as recommended by EAU guidelines) before elective surgery, in order to improve immune function and reduce the risk of infection (27). Such multidisciplinary preoperative optimization is crucial for reducing infection risk in these high-risk patients. Second, for patients with preoperative bacteriuria or pyuria, targeted antibiotic therapy based on preoperative urine culture and sensitivity results is strongly recommended, rather than relying solely on empirical treatment. Even asymptomatic bacteriuria should be considered for eradication prior to surgery, in accordance with EAU guidelines on infection prevention (27). Third, operative time (*OR* = 1.05), as a modifiable surgical factor, warrants particular attention. Although stone size and complexity partially determine the required operative duration, our findings support the implementation of stricter time-consciousness in surgical planning and quality control. The surgical team should be aware that the risk of FUTI increases continuously with prolonged operative time, particularly beyond 75 min (12). This underscores the importance of optimizing surgical efficiency and workflow through adequate preoperative planning (e.g., detailed imaging review), the use of modern high-energy devices and lasers, and consideration of staged procedures or suitable alternatives (such as PCNL) for particularly complex or large stones. Proactive management of operative time is a key component in achieving high-quality surgical outcomes. Fourth, in patients with imaging findings such as perinephric fat stranding or tissue rim sign on CT, which indicate more severe local inflammation and obstruction, surgeons should adjust their surgical strategy accordingly. For instance, anticipating increased surgical difficulty, an experienced surgeon should be assigned, and efforts should be made to minimize operative time and reduce irrigation pressure and duration. Finally, a risk stratification model should be established: a simple preoperative risk assessment tool based on the robust potential factors derived from this meta-analysis (e.g., diabetes, renal insufficiency, bacteriuria, CT findings) could be developed in the future to identify high-risk patients and implement more aggressive preventive measures, such as extended prophylactic antibiotic use or intensified postoperative monitoring.

This study has several limitations: (1) The inclusion was restricted to Chinese and English publications, potentially introducing language bias; (2) Some included single-center studies from China may exhibit temporal and geographical overlaps, raising the risk of duplicate inclusion of the same patient population in the pooled analysis; (3) All included literature originated from published studies, which may be subject to selective publication bias (e.g., unpublished negative results); (4) A relatively high proportion of the included studies employed retrospective case-control designs, and the number of studies available for the analysis of each risk factor was limited, which may affect the robustness of our findings and hinder definitive causal inferences; (5) There was substantial unexplained heterogeneity among the included studies, strongly suggesting the presence of numerous study-level or patient-level confounding factors beyond the macro-level factor of language or region. Although we extracted detailed data on antibiotic management and surgical characteristics, the reporting of these parameters was highly inconsistent and often incomplete across studies (Supplementary Tables 4, 5). This precluded meaningful subgroup or sensitivity analyses based on surgical technique or specific antibiotic protocols, which are important potential sources of the substantial unexplained heterogeneity. Furthermore, the definitions of FUTI varied considerably among studies (Supplementary Table 5), which is a major contributor to heterogeneity and a key area for standardization in future research.

## 5 Conclusion

Based on the above analysis, the synthesis of diabetes mellitus, renal insufficiency, preoperative hydronephrosis, elevated preoperative procalcitonin levels, preoperative pyuria, preoperative bacteriuria, perinephric fat stranding, tissue margin sign, operative duration, and stone size constitutes potential independent predictors for FUTI following surgery in patients with urinary calculi. These factors provide an evidence base for developing a preoperative risk stratification model. Future studies should focus on developing and validating clinical tools to identify high-risk patients and guide individualized perioperative management.

## Data Availability

The raw data supporting the conclusions of this article will be made available by the authors, without undue reservation.
