# Supplementary material for: Risk factors for postoperative febrile urinary tract infection in patients with urolithiasis: a meta-analysis
Source: Front Surg. 2026 Mar 2;13:1772261. doi: 10.3389/fsurg.2026.1772261 (PMC12989538; doi:10.3389/fsurg.2026.1772261)
Supplement: Supplementary file 1 [file Supplementaryfile1.docx]

PubMed

#1 (("Urinary Tract Infections"[MeSH Terms]) OR (Urinary tract infection[MeSH Terms]) OR (Infections, Urinary Tract[MeSH Terms]) OR (Infection, Urinary Tract[MeSH Terms]) OR (Tract Infections, Urinary[MeSH Terms]) OR (Tract Infection, Urinary[MeSH Terms]) OR (Urinary Tract Infections[Title/Abstract]) OR (Urinary Tract Infection[Title/Abstract]) OR (Infections, Urinary Tract[Title/Abstract]) OR (Infection, Urinary Tract[Title/Abstract]) OR (Tract Infections, Urinary[Title/Abstract]) OR (Tract Infection, Urinary[Title/Abstract]))

#2 (Febrile[Title/Abstract])

#3 ((Urolithiasis Surgery[Title/Abstract]) OR (Endoscopic Stone Surgery[Title/Abstract]))

#4 (("Risk Factors"[MeSH Terms]) OR (Risk factor[MeSH Terms]) OR (Factor, Risk[MeSH Terms]) OR (Risk Factors[Title/Abstract]) OR (Risk factor[Title/Abstract]) OR (Factor, Risk[Title/Abstract]))

#5 #1 AND #2 AND #3 AND #4

Embased

#1 **'**urinary tract infection'/exp

#2 'urinary tract infection':ti

#3 (('infection of the urinary tract':ti OR 'lower urinary tract infection':ti OR 'urinary infection':ti OR 'urinary tract infections':ti OR 'urine infection':ti OR 'urine tract infection':ti OR 'urologic infection':ti OR 'urological infection':ti OR uti:ti) AND 'urinary tract infection':ti OR utis:ti) AND 'urinary tract infections':ti OR 'urinary tract infection':ti

#4 'endoscopic stone surgery' OR (endoscopic AND ('stone'/exp OR stone) AND ('surgery'/exp OR surgery))

#5 'urolithiasis surgery' OR (('urolithiasis'/exp OR urolithiasis) AND ('surgery'/exp OR surgery))

#6 'risk factor'/exp

#7 'risk factor':ti

#8 'relative risk':ti OR 'risk factors':ti OR 'risk factor':ti

#9 #1 OR #2 OR #3

#10 #4 OR #5

#11 #6 OR #7 OR #8

#12 #9 AND #10 AND #11 AND [english]/lim AND ([adult]/lim OR [aged]/lim)

#13 postoperative

#14 #12 AND #13

Cochrane Library

#1 MeSH descriptor: [Urolithiasis] explode all trees

#2 MeSH descriptor: [Urinary Tract infections] explode al trees

#3 MeSH descriptor: [Risk Factors] explode all trees

#4 (Lithiasis. Urinary):ti,ab,kw OR (Urinary Lithiasis):ti,ab,kw

#5 (Infection, Urinary Tract):ti,ab,kw OR (Tract Infections, Urinary):ti,ab,kw OR (Tract Infection, Urinary):ti,ab,kw OR (Infections, Urinary Tract):ti,ab,kw OR (Urinary Tract Infection):ti,ab,kw

#6 (Risk Factor):ti,ab,kw OR (Factor, Risk):ti,ab,kw

#7 #1 OR #4

#8 #2 OR #5

#9 #3 OR #6

#10 (postoperative):ti,ab,kw

#11 #7 AND #8 AND #9 AND #10

CBM

(("尿路结石"[不加权:扩展]) OR ("尿路结石"[标题:智能])) AND (("危险因素"[不加权:扩展]) OR ("危险因素"[标题:智能])) AND ("术后尿路感染"[标题:智能])

CNKI

（主题：尿路结石术后）AND（主题：尿路感染）AND（主题：危险因素 + 危险因素分析）

WanFang Data

主题:(尿路结石术后) and 主题:(尿路感染) and 主题:(危险因素 or 危险因素分析)

VIP

M=(尿路结石术后) AND M=(尿路感染) AND M=(危险因素 OR 危险因素分析)
