# Supplementary material for: Risk factors for postoperative febrile urinary tract infection in patients with urolithiasis: a meta-analysis
Source: Front Surg. 2026 Mar 2;13:1772261. doi: 10.3389/fsurg.2026.1772261 (PMC12989538; doi:10.3389/fsurg.2026.1772261)
Supplement: Supplementary file 2 [file Table1.docx]

| Reviewer 1 | Reviewer 2 | | | Total |
| --- | --- | --- | --- | --- |
|  | Exclude | Include | Unclear |  |
| Exclude | 49 | 1 | 1 | 51 |
| Include | 1 | 16 | 0 | 17 |
| Unclear | 0 | 0 | 0 | 0 |
| Total | 50 | 17 | 1 | 68 |

Table S1 The inter-rater agreement for reading the title and abstract

Kappa score: 0.92 (0.85-0.99)
