# Supplementary material for: Risk factors for postoperative febrile urinary tract infection in patients with urolithiasis: a meta-analysis
Source: Front Surg. 2026 Mar 2;13:1772261. doi: 10.3389/fsurg.2026.1772261 (PMC12989538; doi:10.3389/fsurg.2026.1772261)
Supplement: Supplementary file 3 [file Table2.docx]

Table S2 The inter-rater agreement for full-text review

| Reviewer 1 | Reviewer 2 | | | Total |
| --- | --- | --- | --- | --- |
|  | Exclude | Include | Unclear |  |
| Exclude | 1 | 0 | 0 | 1 |
| Include | 0 | 16 | 0 | 16 |
| Unclear | 0 | 0 | 0 | 0 |
| Total | 1 | 16 | 0 | 17 |

Kappa score: 1.00 (1.00-1.00)
