# Supplementary material for: Risk factors for postoperative febrile urinary tract infection in patients with urolithiasis: a meta-analysis
Source: Front Surg. 2026 Mar 2;13:1772261. doi: 10.3389/fsurg.2026.1772261 (PMC12989538; doi:10.3389/fsurg.2026.1772261)
Supplement: Supplementary file 4 [file Table3.docx]

**Table S3 Results of the quality assessment using the newcastle‒ottawa scale**

| **First author, Year** | **Study type** | **Selection4** | **Comparability2** | **Exposure/Outcome3** | **Final score** |
| --- | --- | --- | --- | --- | --- |
| Mitsuzuka 2016 | Cohort study | **** | * | ** | 7 |
| Itami  2021 | Case-control study | **** | * | ** | 7 |
| Lin  2021 | Case-control study | **** | * | *** | 8 |
| Koterazawa 2024 | Cohort study | **** | * | ** | 7 |
| Kim  2020 | Case-control study | **** | * | *** | 8 |
| Kim  2018 | Cohort study | **** | * | ** | 7 |
| Kim  2021 | Cohort study | **** | * | ** | 7 |
| Senel  2024 | Case-control study | **** | * | *** | 8 |
| Xu  2024 | Case-control study | **** | * | *** | 8 |
| Ma  2023 | Case-control study | **** | * | ** | 7 |
| Zhou  2024 | Case-control study | **** | * | *** | 8 |
| Qian  2023 | Case-control study | **** | * | ** | 7 |
| Zhang  2022 | Case-control study | **** | * | ** | 7 |
| Lu  2021 | Case-control study | **** | * | ** | 7 |
| Xu  2024 | Case-control study | *** | * | ** | 6 |
| Chen  2024 | Case-control study | **** | * | ** | 7 |

*study quality according to the Newcastle‒Ottawa Scale across three domains: Selection of study groups (max 4 stars, ****), Comparability of groups (max 2 stars, **), Outcome/Exposure assessment (max 3 stars, ***)
